# A Practical Treatise on the Functions and Diseases of the Unimpregnated Womb

**Published:** 1841-01-01

**Authors:** 


### A Practical Treatise on the Function and Diseases of the Unimpregnated Womb. Illustrated by Plates, &c. with a Chapter on Leucorrhœa, Fluor Albus, or Weakness. By *Charles Waller,* M.D.

The subject of uterine disorders has been so frequently and voluminously discussed of late years that one would suppose there could be no demand for another work of the kind, which merely repeats or comprises the opinions expressed in those which have gone before it: however, the subject is so important an one, that a practical treatise on some of the most common affections may still be a desideratum.

Dr. Waller has endeavoured to supply this by the book now under notice, which is made up of a series of lectures delivered by the author at the "Aldersgate-street School," and has been published in the present form, to give a more continuous, and connected view of the matters treated of, than was possible when each lecture was scattered among the pages of a weekly periodical.

The work however is not to be considered as a complete dissertation on the diseases of the unimpregnated womb, for it is only a very condensed description of a few of them, the author declaring in his preface how fully he is impressed with the force of the aphorism "μεγα βιβλιον μεγα κακον:" Now though we are willing to admit that a great book may be a great evil, yet it by no means follows that a small one must be the contrary, and in the present instance Dr. Waller has been so far led astray by his reverence for this dogma, as to omit the mention of many facts which would have greatly added to the utility of his plain and practical descriptions, without rendering them laboured, or diffuse.

The arrangement, or division of the subject, is a very simple one, and perhaps for all practical purposes, is as useful as the more complicated classifications adopted by some of the writers in this class of diseases; thus the only division of it is into "Disorders of the Menstrual Function" and "Diseases of the Unimpregnated Womb." The former of course including the various kinds of catamenial disturbance, and the latter, some of the principal organic lesions to which the uterus is obnoxious:—the whole subject being discussed in the following order, viz:—

1. The Function of the Unimpregnated Uterus.
2. The Disorders of this Function.
3. The Period of Female Existence designated the "Turn of Life," and
4. The Diseases of the Womb itself.

The Chapter on the "Function of the Unimpregnated Womb" presents us with no new facts, and consists of the usual observations on the periodicity of the menstrual discharge; the average age at which it first appears; the local and general changes which characterise this epoch of life; the particular part which furnishes the discharge, &c.

The author takes occasion to repudiate the idea of the menstrual fluid being possessed of any "malignant property;" but after all, it is not quite so certain that the old writers were altogether wrong in maintaining the affirmative of the question: that they *exaggerated* the mischief is certain, but that it often causes præputial excoriations to put on the characters of irritable, and to become troublesome ulcers, is a fact of which we at least have no doubt.

Emansio, and suppressive mensium, the two forms of amenorrhœa, are discussed together with chlorosis: under the first head are some brief practical remarks on retained menses from imperforate hymen, and occlusion of the os uteri: also on emansio arising from faulty development, as absence of the ovaries, &c.; but there is some confusion caused by designating "chlorosis" as one of the conditions of "Emansio," whereas this particular state is much oftener met with during temporary "suppression;" nor, in point of fact, is it necessarily the concomitant of either, for it not unfrequently happens, that chlorotic girls menstruate regularly, and *properly*; without doubt the general rule is, that if there be any menstrual discharge in these cases of chlorosis, it is scanty and pale-coloured, but there are many exceptions to it notwithstanding. Dr. Waller very justly insists on the propriety of considering the absence of the catamenia in such cases, as an effect of the generally deranged health, and not as a cause of it: indeed this opinion needs no other proof of its correctness than the fact, that in many instances the menses do not flow for several months after the general symptoms of chlorosis have vanished, and the patient has been quite restored to health and strength.

We should not imagine that any well-informed practitioner would be likely to mistake chlorosis for "pulmonary disease:" still we are warned of the danger at page 22:—the usual absence of the catamenia in tubercular phthisis, might have been mentioned in an enumeration of the symptoms common to both.

In his treatment of chlorosis our author adopts the one generally employed. He is opposed to the use of emetics, and recommends the combination of stimuli with tonic remedies in some cases: he gives a formula for pills on this principle at p. 25, to the efficacy of which we can speak favorably. To the employment of the forcing remedies called emmenagogues, Dr. Waller is stoutly opposed: in fact he never loses an opportunity of inveighing against the practice, or of shewing the absurdity of it; his remarks on this point are always judicious, and the following sentence will prove how laudably earnest he is in the matter. "The reader is earnestly implored not so far to forget rational principle as to be induced to have recourse to that empirical practice which consists in the administration of those stimulating and forcing remedies called emmenagogues."

Again, when speaking of the treatment of emansio mensium attended with plethora, for which he recommends periodical bleedings—he says, "The use of stimulating emmenagogue medicines can in nowise be justified, as the non-appearance of the discharge is the result of an action of the menstrual vessels, very analogous if not actually amounting to inflammation."

The remarks on sudden suppression are good and concise; but the disadvantages of a small book, which we before hinted at, are apparent in such sentences as the following. "Should month after month pass away without the restoration of the secretion, it will in all probability be found that other organs besides the womb are disordered, and these will then require particular attention."

Now we think it is not unreasonable to wish that a book especially intended as a practical guide for students, should have been a little more explicit, as to these probably diseased organs. The subject of menorrhagia is briefly but ably discussed: Dr. Waller uses the term in its most extended sense, including all undue sanguineous discharges from the unimpregnated womb, of course distinguishing those which contain " integral blood," from those which are only menstrual fluid in excess.

Profuse menstruation is however, as our author justly observes, comparatively rare, and even then, it is not necessarily to be considered as a disease. In the active form of menorrhagia Dr. Waller depends mainly on blood-letting and laxatives: after which he recommends the nitrate of potass in doses from gr. xv. to xxv. " well diluted in barley water," as a most effective depressing agent; he very wisely questions the utility of mercury, and is no friend to the ergot. His description of the general symptoms of " passive menorrhagia" is as follows.

" With this hemorrhage there is a rapid reduction of the little strength previously existing; the countenance is pallid, in some cases asuming an almost bloodless appearance; the pulse hurried and feeble; the extremities, and sometimes the whole surface of the body, cold; there is a weight and pain in the head, particularly over the eyebrows and forehead; a distressing sensation of faintness and giddiness, and occasionally nausea and vomiting; laborious respiration is also a frequent attendant on the more severe and dangerous forms of passive menorrhagia." 51.

All of the remarks in this division of the subject are sound and practical; but we should imagine that the cases of this affection, where it will be necessary to " plug the vagina," must be very rare indeed; particularly as it cannot be forgotten that the discharge is, at least in part, a secretion! however, the author mentions a case which occurred in his own practice, where this plan was obliged to be resorted to, and was quite successful, even after the failure of Dr. Haighton's astringent uterine injections. (vide p. 55.)

The chapter on the " *Turn of Life*" is rather bare and meagre; a few observations are made relative to the frequency of uterine, and mammary diseases at this period, which the author explains thus—" This opinion is decidedly erroneous: the true explanation of the reason why an advance of disease is so frequently noticed at the " turn of life" is simply this, that the constitution, or the parts disposed to morbid action, are not now, as heretofore, relieved by the local determination and secretion."

The subject of Sterility or " Barrenness" is dismissed in three pages: Dr. Waller does not seem to know any more about it than his neighbours.

The second part of the " Treatise" is devoted to the consideration of the " *Diseases* of the Unimpregnated Womb," and the author commences the subject by a few remarks on the importance of tracing symptoms to their original cause, since the curable and the incurable diseases have often a great similarity in this respect.

On the Structure and Pathology of Hydatids, we have a longer account than usual; it is evidently a favorite subject, and two out of the six plates belonging to the work exhibit specimens of this disease. On the question of their ever being produced " sine copula maris," the author gives no decided opinion; he has never known them in unmarried females, " although he should feel great hesitation in pronouncing this to be an impossible occurrence."

Neither does he coincide in the opinion that these vesicular growths are morbid enlargements of the chorion, and to shew that they are not so, he refers to plate 2, which represents a preparation from his own museum, where the uterus being thickened and enlarged, is studded throughout its substance with little hydatid vesicles, a few of which project into the cavity covered by, and behind the mucous membrane: certainly the author has a right to say with regard to this:—" these appearances could not possibly be caused by any morbid alteration of the chorion."

The following quotation will give an idea of the author's opinions on the nature and origin of malignant disease.

" We believe that this and most other species of malignant disorganizations of the uterus arises from the same exciting cause: that inflammation is the *fons et origo mali;* that it is not essentially specific in its character, but observes the same laws, and yields to the same treatment as any ordinary case of inflammation; that the specific character which the disease afterwards assumes depends not upon the application of any peculiar exciting cause, but that the character of such disorder is determined by the tendency which exists in the individual constitution; and, lastly, that, therefore, the same immediate cause will produce in one, common inflammation; in a second, malignant ulcer; in a third, tubercle; in a fourth, cancer; and so on." 133.

This very naturally introduces the subject of Cancer Uteri. In the present treatise the term carcinoma is applied to the ulcerative stage, and the term " scirrhus" only to that hardened enlargement which precedes it.

The opinion already expressed with regard to the first cause of malignant disease is reiterated now: inflammatory action, and that not of a specific kind, but exerting itself in a peculiar constitution, is again stated as the origin of this dreadful malady; and Dr. Waller is very earnest in directing the attention of practitioners to the state of the uterus in all complaints of women at the period of menstrual cessation, lest from an unfortunate delicacy on the part of the patient, who will not often be induced to speak of pains referrible to those parts, this insidious disease should grow unheeded. He is strongly of opinion, that this first stage of " stony hardness" is recoverable from by rigid and long-continued antiphlogistic treatment; and at page 162 he mentions a case cured when in this condition, by repeated spontaneous hemorrhage from the uterus.

The plan of drawing blood immediately from the cervix and os uteri, by introducing leeches there enclosed in a tube is not encouraged by Dr. Waller: strangely enough, he thinks their application to the vulva and the os externum a " preferable method."

No doubt the latter mode is the easier of execution, as the former one requires the personal attention of a medical attendant, or of one trained to the art; but we think there can be no rational doubt which of the two is the more effective.

The treatment recommended in this stage of " scirrhus," besides the local abstraction of blood, consists of the usual palliative measures, which after being enumerated are thus disposed of.

" These, then, are the remedial means to be employed in scirrhus of the uterus; and we conclude our observations on the subject by imploring the reader not to imagine, because the disease is usually fatal, that therefore nothing can ever be effectually accomplished for the patient's relief. We confidently re-state our conviction, that much may be effected *at the commencement,* not only in the way of palliation, but for the eventual arrest of its progress ; the insurmountable difficulties so frequently met with being the result of delay, and this manifestly arising from the slight, and to the patient unimportant, symptoms which indicate its first and only curable stage." 166.

In the ulcerative stage of carcinoma, Dr. Waller, like every body else, has little to recommend, as then " all hopes of cure must be abandoned." He says, and says justly, that nothing but opium will deaden the agonizing pain of the miserable patient; and that the doses of the remedy are not to be regulated by counting the number of grains given, but by observing the effects it produces.

On the question of excision of the uterus, as a last resource, the author quotes Dr. Blundell's results of the four cases operated on by this accomplished obstetrician! he admits that they give no encouragement for future attempts, and in spite of M. Lisfranc, there are not many who hold a different opinion.

We cannot conclude this analysis of Dr. Waller's book without alluding to his total silence on the diagnostic signs derivable from the touch and the speculum. The latter instrument is only mentioned once in the whole book, and then only with a view of forbidding its employment on the score of indelicacy. We have always reprobated the indiscriminate use of it so disgustingly insisted on by some French writers ; but there are cases in which it must be used, and as an aid to diagnosis, it ought at least to be mentioned as useful in a practical treatise on uterine diseases. The same may be said of manual examinations " per vaginam ;" this mode is not open to the same amount of objection as the other, and the " practical treatise" of Dr. Waller would not have been the worse for a few practical remarks on this important and very difficult subject.

The six lithographic drawings which embellish the book present curious specimens of the diseases which they very cleverly illustrate.

